# The EUPEMEN (EUropean PErioperative MEdical Networking) Protocol for Acute Appendicitis: Recommendations for Perioperative Care

**DOI:** 10.3390/jcm13226943

**Published:** 2024-11-18

**Authors:** Orestis Ioannidis, Elissavet Anestiadou, Jose M. Ramirez, Nicolò Fabbri, Javier Martínez Ubieto, Carlo Vittorio Feo, Antonio Pesce, Kristyna Rosetzka, Antonio Arroyo, Petr Kocián, Luis Sánchez-Guillén, Ana Pascual Bellosta, Adam Whitley, Alejandro Bona Enguita, Marta Teresa-Fernandéz, Stefanos Bitsianis, Savvas Symeonidis

**Affiliations:** 1 Fourth Department of Surgery, Medical School, Faculty of Health Sciences, Aristotle University of Thessaloniki, General Hospital “George Papanikolaou”, 57010 Thessaloniki, Greece; elissavetxatz@gmail.com (E.A.); sbitsiani@gmail.com (S.B.); simeonidissavvas@yahoo.com (S.S.); 2 Institute for Health Research Aragón, 50009 Zaragoza, Spain; jramirez@unizar.es (J.M.R.); jmte-zubieto@hotmail.com (J.M.U.); anapascual689@gmail.com (A.P.B.); secretariagerm@gmail.com (A.B.E.); mteresa@iisaragon.es (M.T.-F.); 3 Department of Surgery, Faculty of Medicine, University of Zaragoza, 50009 Zaragoza, Spain; 4 Department of Surgery, Azienda Unità Sanitaria Locale Ferrara—University of Ferrara, 44121 Ferrara, Italy; n.fabbri@ausl.fe.it (N.F.); cvfeo@unife.it (C.V.F.); antonio.pesce@ausl.fe.it (A.P.); 5 Department of Anesthesia, Resuscitation and Pain Therapy, Miguel Servet University Hospital, 50009 Zaragoza, Spain; 6 Department of Plastic Surgery, Second Faculty of Medicine, Charles University and Motol University Hospital, 150 06 Prague, Czech Republic; kristina.rosetzua@gmail.com; 7 Department of Surgery, Universidad Miguel Hernández Elche, Hospital General Universitario Elche, 03203 Elche, Spain; arroyocir@hotmail.com (A.A.); drsanchezguillen@gmail.com (L.S.-G.); 8 Grupo Español de Rehabilitación Multimodal (GERM), 50009 Zaragoza, Spain; 9 Department of Surgery, Second Faculty of Medicine, Charles University and Motol University Hospital, 150 06 Prague, Czech Republic; kocian.cz@gmail.com; 10 Department of Surgery, University Hospital Kralovske Vinohrady, 100 34 Prague, Czech Republic; whitley.adam@gmail.com

**Keywords:** acute appendicitis, appendectomy, appendicitis diagnosis score, appendicitis guidelines, laparoscopic appendectomy, perioperative care, surgical rehabilitation, EUPEMEN project, enhanced recovery after surgery, training and dissemination

## Abstract

**Background/Objectives:** Acute appendicitis (AA) is one of the most common causes of emergency department visits due to acute abdominal pain, with a lifetime risk of 7–8%. Managing AA presents significant challenges, particularly among vulnerable patient groups, due to its association with substantial morbidity and mortality. **Methods**: The EUPEMEN (European PErioperative MEdical Networking) project aims to optimize perioperative care for AA by developing multidisciplinary guidelines that integrate theoretical knowledge and clinical expertise from five European countries. This study presents the key elements of the EUPEMEN protocol, which focuses on reducing surgical stress, optimizing perioperative care, and enhancing postoperative recovery. **Results**: Through this standardized approach, the protocol aims to lower postoperative morbidity and mortality, shorten hospital stays, and improve overall patient outcomes. The recommendations are tailored to address the variability in clinical practice across Europe and are designed to be widely implementable in diverse healthcare settings. **Conclusions**: The conclusions drawn from this study highlight the potential for the EUPEMEN protocol to significantly improve perioperative care standards for AA, demonstrating its value as a practical, adaptable tool for clinicians.

## 1. Introduction

Acute appendicitis (AA) is the most commonly encountered surgical cause of lower abdominal pain leading to emergency department visits, with a lifetime risk of 7–8% in the United States for the years 1979–1984, according to the National Hospital Discharge Survey data [1,2]. The postoperative complication rate is approximately 10–19% for uncomplicated AA, which rises to 30% in cases of complicated AA [1]. The advent of laparoscopic appendectomy has resulted in reduced postoperative wound pain and infection rate, a lower complication rate, early food tolerance, and shorter hospital stay [3].

Public health principles are central to the development and implementation of protocols aimed at improving surgical outcomes on a population level [4]. Public health strategies focus on preventing disease, prolonging life, and enhancing healthcare levels through organized efforts and informed choices made by society, organizations, public and private sectors, communities, and individuals [5]. In the context of acute appendicitis, a condition with significant morbidity and mortality, public health interventions aim to standardize care, reduce disparities in health outcomes, and ensure that all patients receive evidence-based, high-quality perioperative management [6]. Public health interventions in perioperative care include the development and dissemination of clinical guidelines, the training of healthcare professionals, and the monitoring of outcomes to ensure adherence to best practices. Implementing these interventions effectively requires a multi-faceted approach that includes education, the establishment of standardized protocols, and ongoing evaluation and quality improvement initiatives [5]. The EUPEMEN project has been developed with respect to the abovementioned principles, with the intent to harmonize perioperative care across Europe by providing a standardized protocol that is accessible, evidence-based, and adaptable to various healthcare settings.

The existing and well-established Enhanced Recovery After Surgery (ERAS) protocols have demonstrated significant improvements in patient outcomes by minimizing surgical stress, reducing postoperative complications, and shortening hospital stays. However, the universal application of these protocols remains a challenge due to variability in healthcare resources, provider practices, and patient populations [7,8]. The EUPEMEN protocol for acute appendicitis builds on the principles of ERAS by offering a more tailored approach that addresses the specific needs of patients undergoing appendectomy. By focusing on the basic elements of the protocol, our aim is to provide a framework that can be easily implemented and adapted by healthcare providers, thus ensuring the broader adoption and more consistent application of best practices across different settings.

Enhanced recovery after surgery protocols aim at reducing the stress response after surgery, postoperative complications, and mortality by optimizing perioperative care [9]. The cornerstone elements of ERAS include preoperative counseling and rehabilitation, nutrition assessment and support, multimodal opioid-sparing analgesia, and early mobilization [10]. ERAS programs generally consist of three phases: preoperative, intraoperative, and postoperative, and are based on close interdisciplinary collaboration between different specialties, including surgeons, anesthesiologists, nurses, and other paramedical health professionals [11].

Over the last years, numerous guidelines have been published regarding major issues of considerable controversy, including the timing of appendectomy, the safety of the wait-and-watch in-hospital approach, the role of interval appendectomy after conservative treatment, stump management, and the management of incidental intraoperative findings [12]. The emersion of ERAS programs has led to the radical improvement of postoperative outcomes in elective surgery [13]. However, the literature is generally scarce regarding the results of multimodal surgical rehabilitation guidelines in the emergency setting, and especially in the management of AA [14]. In addition, the full implementation of ERAS principles in emergency cases is vague and impaired by conventional preferences and practices.

In order to achieve the widespread application of the ERAS principles among healthcare facilities in Europe, the EUPEMEN (European PErioperative MEdical Networking) project was conceived and implemented by five partners with strong medical and academic backgrounds, working in academic hospitals in four different European countries. The aim of the EUPEMEN recommendations was to utilize and combine theoretical knowledge and clinical experience in order to achieve the widespread implementation of ERAS protocols regarding specific fields of surgery in Europe. The result was a standardized open-access educational ERAS protocol that is available through a learning website platform and helps the implementation of evidence-based ERAS protocols in a standardized way among European healthcare facilities. The EUPENEM Project received support by the ERASMUS+ program (agreement number 2020-1-ES01-KA203-082681, date of approval: 21 September 2020) of the European Union (EU).

It should be highlighted that, while AA is a commonly encountered condition in surgical practice, its perioperative management—especially in emergency contexts—lacks a standardized, comprehensive approach across diverse healthcare systems. The EUPEMEN project introduces a pioneering, structured ERAS protocol specifically designed for AA, offering a detailed, step-by-step framework that addresses the distinct needs of AA in emergency settings. In contrast to general ERAS guidelines, which are primarily focused on elective procedures, the EUPEMEN protocol provides tailored, evidence-based recommendations for AA, with practical applications that extend to resource-limited settings, including low- and middle-income countries. This novel approach promotes consistency in perioperative care, supporting improved outcomes and the wider implementation of ERAS principles for AA. The aim of this study is the presentation of the basic elements of the proposed protocol for AA management within the EUPEMEN project.

## 2. Methods

### 2.1. Protocol Development

The EUPEMEN project was run by five collaborators from university hospitals originating from four different European countries. The project was launched by Fundación Instituto de Investigación Sanitaria Aragón-IISA (Spain) with partners from Azienda Unità Sanitaria Locale Ferrara—AUSLFE (Italy), Univerzita Karlova—CUNI (Czech Republic), Universidad Miguel Hernández de Elche—UMH (Spain), and “G. Papanikolaou—GPAP” General Hospital of Thessaloniki (Greece). These institutions are recognized leaders in the fields of surgery and medical research, with a strong focus on enhancing patient outcomes through evidence-based protocols. More specific details are provided below.

- **Fundación Instituto de Investigación Sanitaria Aragón (IISA), Spain**: As the coordinator of the EUPEMEN project, IISA plays a central role in advancing medical research and clinical practice. This institute is well known for its leadership in surgical rehabilitation and the development of multimodal recovery programs. Their extensive experience in coordinating large-scale international projects ensures that the EUPEMEN protocol is both comprehensive and grounded in the latest evidence-based practices. In particular, Dr. Jose M. Ramirez is a leading figure in surgical rehabilitation and multimodal recovery programs. He is the current president of GERM, the Spanish Multimodal Rehabilitation Group. He brings extensive experience in managing large-scale international research initiatives and ensures that the perioperative care protocols are rooted in rigorous scientific evidence and practical clinical experience. Dr. Javier Martínez Ubieto, a leading researcher in the Anesthesiology department at Miguel Servet Hospital, has extensive experience in multimodal rehabilitation. He played a key role in developing and implementing the Multimodal Rehabilitation in Radical Cystectomy program, which successfully reduced morbidity and hospital stays. His work, highlighted in Dr. Sonia Maria Ortega Lucea’s doctoral thesis and published in Actas Urológicas Españolas, received the Teaching Innovation award for significantly improving healthcare practices [15].
- **Azienda Unità Sanitaria Locale di Ferrara (AUSL Ferrara), Italy:** AUSL Ferrara is a key partner in the EUPEMEN project, known for its strong integration of research into clinical practice. The institution specializes in laparoscopic surgery and enhanced recovery after surgery (ERAS) protocols, bringing valuable practical insights to the development of the EUPEMEN guidelines, particularly in tailoring them to real-world surgical settings. In particular, Dr. Carlo Feo, chief of the General Surgery Unit and deputy head of the Department of Surgery, pioneered an Enhanced Recovery Program (ERP) at the Saint Anna University Hospital in 2011, establishing a multidisciplinary team to implement high-evidence perioperative care protocols. He later extended the ERP to colorectal surgery in a rural district served by AUSL Ferrara in 2017.
- **Charles University, Prague, Czech Republic:** Charles University, with its rich tradition of medical education and research, is a leader in emergency surgery and perioperative management. The university’s involvement in the EUPEMEN project ensures that the protocol benefits from cutting-edge surgical techniques and a deep understanding of the challenges in managing acute surgical conditions across different healthcare environments. In particular, Dr. Petr Kocián, a consultant surgeon at the Department of Surgery at Charles University’s Second Faculty of Medicine, played a key role in establishing the ERAS protocol for colorectal surgery. Specializing in mini-invasive colorectal surgery, he is an active member of leading organizations such as the European Society for Coloproctology and the European Association for Endoscopic Surgery. Dr. Kocián has authored several research articles on colorectal surgery published in peer-reviewed journals.
- **Universidad Miguel Hernández de Elche (UMH), Spain:** UMH is known for its strong emphasis on surgical research and enhanced recovery pathways. The university’s expertise in both elective and emergency surgeries has significantly contributed to the adaptability and effectiveness of the EUPEMEN protocol in various clinical scenarios. In particular, Dr. Arroyo’s expertise in both elective and emergency surgeries, combined with his strong focus on surgical research, has significantly contributed to the adaptability and effectiveness of the EUPEMEN protocol. His work ensures that the guidelines are practical and applicable to a wide range of clinical scenarios.
- **Papanikolaou General Hospital of Thessaloniki, Aristotle University of Thessaloniki, Greece:** The Fourth Department of Surgery at Aristotle University is renowned for its excellence in surgical education and emergency care. Their substantial experience in developing and applying evidence-based protocols for acute conditions, including acute appendicitis, has been integral to the formulation of the EUPEMEN guidelines. In particular, Dr. Ioannidis is a respected leader in surgical education and emergency care. His substantial experience in developing evidence-based protocols for acute conditions, such as acute appendicitis, has been integral to the formulation of the EUPEMEN guidelines, ensuring they are both scientifically sound and clinically relevant.

The fundamental aim of the EUPEMEN project is to provide healthcare professionals, including surgeons, anesthesiologists, nurses, and allied health professionals, with a set of recommendations for the best standards of surgical rehabilitation through the collaboration of multiple specialists, based on the clinical and theoretical background of the five partners. It should be highlighted that the patient target groups of EUPEMEN protocol are adults and elderly people, who often present with more complex comorbidities and different physiological responses to treatment compared to pediatric population. This focus is reflected in the expertise of the protocol’s development team, which comprised only general surgeons with theoretical and clinical experience in adult populations.

The European consensus panel performed a non-systematic review of the available literature regarding guidelines for perioperative management in AA and appendectomy. In 2015, the Clinical Pathway for Intensified Recovery in Abdominal Surgery (Via RICA) protocol for abdominal surgical procedures was established based on the consensus of numerous scientific societies in an attempt to enhance uncomplicated postoperative recovery and patient safety and to improve postoperative outcomes [16]. Later, in 2021, the Via RICA protocol was updated to standardize the surgical care principles according to intensified recovery protocols in numerous other surgical areas other than abdominal [17]. It should be highlighted that the Via RICA protocol constitutes a comprehensive systematic review of the existing knowledge on perioperative care and enhanced recovery protocols has already been conducted as part of the development of the Via RICA protocol, which forms the basis of the EUPEMEN project. This systematic review thoroughly assessed and ranked the evidence related to each aspect of perioperative care, ensuring that the recommendations in the EUPEMEN protocol are grounded in the highest levels of available evidence. Given that this process has been extensively addressed in Via RICA, the EUPEMEN protocol for AA focuses on the practical application and specific adaptations of these guidelines rather than reiterating the systematic review itself. The consensus for the EUPEMEN protocol was reached through a structured, multi-phase process involving a multidisciplinary panel of experts, including surgeons, anesthesiologists, and other allied health professionals, from five university hospitals across four European countries. These experts were selected based on their clinical experience, academic contributions, and involvement in national healthcare programs related to surgical care. The process was designed to ensure that the protocol is both evidence-based and reflective of the collective expertise of the participating specialists. Based on the Via RICA protocol and on the articles chosen for review, the consensus formed as initial drafting a list of suggestions for optimizing perioperative care after appendectomy for AA and for reducing clinical practice variability among centers. This review provided the foundational knowledge and identified key areas of variability in clinical practice that needed to be addressed. This list was further distributed to scientists of collaborative employment [18]. This method involved several rounds of anonymous surveys where panel members provided their input on various aspects of the protocol. After each round, the responses were aggregated and shared with the group, allowing for the iterative refinement of the protocol. Following the Delphi rounds, three face-to-face meetings were held to discuss the aggregated results, resolve any remaining disagreements, and finalize the protocol. These meetings were crucial for addressing complex issues that required in-depth discussion and collaboration among the experts. The final version of the protocol was validated by all participating experts. Each expert had the opportunity to review the protocol, suggest last-minute changes, and formally approve the final document. The protocol was then distributed to additional clinicians within the participating hospitals for further feedback, ensuring that it was practical and applicable in real-world settings. This rigorous consensus process, combining a literature review, iterative Delphi rounds, and collaborative meetings, ensured that the EUPEMEN protocol is both comprehensive and widely supported by experts in the field.

**Figure 1:**
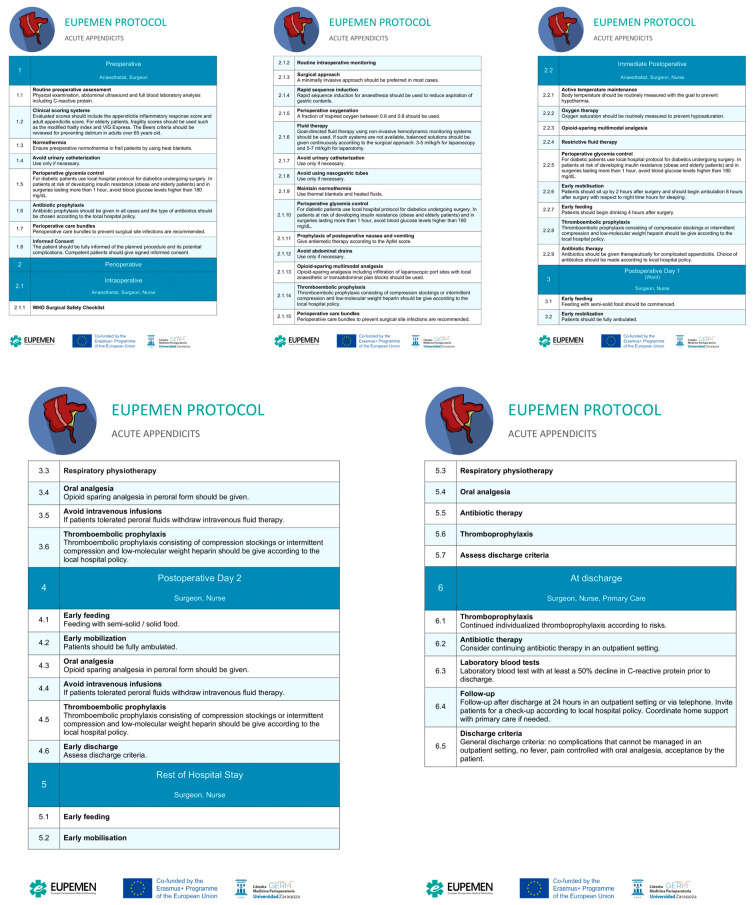
The EUPEMEN protocol in surgery for AA.

The EUPEMEN protocol for AA surgical management presents the following structure: (1) a preoperative phase, involving the surgeon and the anesthetist; (2) the perioperative phase, including the intraoperative and the immediate postoperative phases, as well as possible intensive care unit (ICU) stay, and involving the surgeon, the anesthetist, and the nurse; and (3) the postoperative period, lasting until the end of the end of postoperative day 2 (POD2), the rest of hospital stay, if applicable, and discharge of the patient (Figure 1).

Apart from health professionals directly involved in the care of surgical patients (i.e., surgeons, anesthetists, and nurses), the EUPEMEN protocol also addresses allied professionals responsible for the improvement of perioperative care, such as dietitians, stoma therapists, physiotherapists, geriatricians, radiotherapists, oncologists, and pathologists. The current protocol constitutes an important adjuvant for professional primary healthcare providers involved in the surgical procedure, such as surgeons, anesthetists, and nurses, as well as for healthcare providers involved in the perioperative process, such as physiotherapists or geriatricians, in the case of elderly patients.

### 2.2. Technical Activities

The EUPEMEN project had six main technical projects, which were as follows:

(1) The production and distribution of the *EUPEMEN Multimodal Rehabilitation Manual*, which includes recommendations for six surgical disciplines: (a) esophageal surgery, (b) gastric cancer surgery, (c) liver surgery, (d) bariatric surgery, (e) colorectal surgery, (f) acute appendicitis, and (g) bowel obstruction. The manual is available in five different languages (English, Spanish, Italian, Greek, Czech), and is accessible through the EUPEMEN project website (https://eupemen.eu/eupemen-manuals/, accessed on 1 November 2024).
(2) The EUPEMEN online platform, accessible without charge through the EUPEMEN Learning Website (https://eupemen.eu/, accessed on 1 November 2024), which offers a series of courses for all interventions predicted in terms of enhanced recovery. The online course materials are derived from the RICA Pathway.
(3) The training of the trainers, serving the primary aim of the EUPEMEN project to transfer the principles of perioperative optimization to the future healthcare experts and teachers.
(4) The organization of five multiplier events for promoting and disseminating the aims and content of the EUPEMEN project.
(5) The organization of four international meetings, one per participating country.
(6) The translation of the Spanish manual de Recuperación Intensificada en Cirugía del Adulto (RICA) to the English revised version, entitled “the Recovery Intensification for optimal Care in Adult’s surgery (RICA)” [16]. This project, developed by the Grupo Español de Rehabilitación Multimodal (GERM) and the Spanish Ministry of Health, Social Services, and Equality, has the principal goal of eliminating variability in clinical practice.

Since the time of its conception, the outcomes of the EUPEMEN project have included the development of the EUPEMEN Protocols Training Programme for health professionals; the successful training of 200 multidisciplinary health professionals participating in the perioperative setting; the establishment of one local forum with 40 participants for each EUPEMEN partner; the application of the protocol principles in at least five European hospitals; and the establishment of an educative network with training roles and responsibilities for auditing the correct implementation of the guidelines. The future goals and long-term impact of the project are the improvement in perioperative metrics through and quicker recovery and shorter hospital stay, reduced morbidity and mortality rates, and, consequently, a reduction in the economic burden for the public health system.

### 2.3. Future Research Aims

The EUPEMEN project is committed to continuously advancing perioperative care for acute appendicitis and other surgical conditions. Future research will focus on evaluating the effectiveness and impact of the EUPEMEN protocol across diverse healthcare settings in Europe. This includes conducting multi-center studies to measure key outcomes such as postoperative recovery times, complication rates, and patient satisfaction. Additionally, studies investigating the adaptability of the protocol in different clinical environments, particularly in hospitals with varying levels of resources, to ensure its applicability and efficacy across all settings are planned. In addition, the assessment of the long-term effects of protocol implementation on healthcare costs and resource utilization will be performed.

### 2.4. Objectives for Implementation

The primary objective of implementing the EUPEMEN protocol is to standardize perioperative care for acute appendicitis across Europe, thereby reducing variability in clinical practice and improving patient outcomes. The EUPEMEN project aims to achieve widespread adoption of the protocol through targeted education and training programs for healthcare professionals, including surgeons, anesthesiologists, nurses, and allied health professionals. Additionally, the focus will be on integrating the protocol into existing healthcare systems, ensuring that it is accessible, practical, and sustainable. The long-term goal of the EUPEMEN project is to establish a network of European hospitals that routinely utilize the EUPEMEN protocol, facilitating continuous improvement and knowledge sharing to enhance surgical care across the region.

## 3. Results

### 3.1. The EUPEMEN Protocol in Surgery for AA 

#### 3.1.1. Preoperative Phase

The preoperative phase of the EUPEMEN protocol for AA is mainly performed by the surgeon and the anesthesiologist and begins with the routine preoperative medical assessment of the patient, including physical examination, abdominal ultrasound, and blood sample laboratory tests, including C-reactive protein (CRP) analysis [19,20]. Numerous clinical scores have been proposed for severity evaluation and stratification, as well as an adjuvant for decision making and treatment selection [21]. All adult patients should be assessed with the Appendicitis Inflammatory Response (AIR) Score and the Adult Appendicitis Score (AAS) [22,23]. For elderly patients, a fragility assessment score, such as the Modified Frailty Index and VIG Express, should be included in the preoperative setting, along with an anesthesiologic risk score [24,25,26]. Appendectomy has been proven to be a significant independent risk factor for postoperative delirium in the acute care surgery setting [27]. To address this need, the American Geriatrics Society Beers Criteria should be checked for postoperative delirium avoidance in all patients older than 65 years [28].

Providing and maintaining preoperative normothermia is very important, since it reduces patient discomfort, incidence of wound infections, rate of allogenic blood transfusions, and length of hospital stay [29]. Especially in frail and critically ill patients, the use of heat blankets promotes normothermia by reducing heat loss and increasing patient comfort [30].

Routine urinary catheterization should be avoided as much a as possible in order to prevent healthcare-associated urinary tract infection [31]. People with diabetes mellitus are at increased risk of postoperative complications, prolonged hospitalization, and increased mortality [32]. For this reason, perioperative glycemic control constitutes a crucial issue of perioperative management.

Local hospital protocols for surgical procedures in diabetic patients should be applied, while for patients vulnerable to insulin resistance, such as obese and elderly patients, as well as before procedures with operative time longer than 1 h, blood glucose concentrations should not be higher than 180 mg/dL [33].

Preoperative care bundles have been established as a method of process standardization, decreasing operative variance, and reducing surgical site infections (SSIs) [34]. The EUPEMEN protocol suggests the implementation of perioperative care bundles aiming at the prevention of surgical site infection, combined with antibiotic prophylaxis in all cases, based on the local hospital policy [35,36].

Along with medical assessment, patients should also be thoroughly informed with verbal and written information regarding the procedure, possible complications, and methods to improve recovery after surgery [37]. Signed informed consent should be provided by all competent patients, despite the emergent character of the procedure [38,39].

Despite the fact that the preoperative phase is primarily focused on medical assessments and decisions that are crucial for surgical planning and is often led by surgeons and anesthesiologists, the nursing team also holds an essential role in ensuring patients are comprehensively prepared for surgery. Their responsibilities encompass patient education, where they provide detailed information about the surgical procedure, outline expectations for the perioperative period, and address any questions or concerns the patient may have [40]. Nurses also assist in the collection and verification of critical preoperative data, such as medical history and current medication use, ensuring that all necessary protocols, such as fasting and medication adjustments, are adhered to. Moreover, they offer psychological support, helping to mitigate patient anxiety and foster a sense of preparedness. This involvement is crucial in optimizing surgical outcomes and facilitating a smooth transition through the perioperative process.

#### 3.1.2. Intraoperative Phase

During the intraoperative stage of AA management, the implementation of the World Health Organization (WHO) Surgical Safety Checklist is necessary, since it promotes patient safety [41,42]. The surgeon, the anesthesiologist, and the nurse hold a crucial role during this phase [43].

The main responsibility of the anesthesiologist, according to the principles of the EUPEMEN protocol, is maintenance of routine intraoperative monitoring. The latter includes non-invasive blood pressure evaluation; electrocardiography monitoring via an electrocardiogram with five leads, with V5 and DII being recommended [44]; the measurement of the fraction of inspired oxygen (FiO_2_); oxygen saturation via pulse oximetry; capnography (EtCO_2_); and providing a perioperative oxygen supply with a fraction of inspired oxygen of between 0.6 and 0.8 [45]. In addition, the monitoring of core body temperature and normothermia maintenance with use of thermal blankets and heated fluids is necessary [46]. Perioperative glucose level monitoring for diabetics, according to local hospital protocols, and the maintenance of blood glucose concentration in levels lower than 180 mg/dL in patients at risk of developing insulin resistance and in procedures longer than 1 h remain important points of attention, even during the intraoperative phase [47]. Goal-directed fluid therapy (GDT) is a basic element of the ERAS protocol, aimed at minimizing complications originating from perioperative fluid imbalance [48]. GDT, with the application of non-invasive hemodynamic monitoring systems, is advised. In the absence of such systems, the continuous administration of balanced solutions depending on the surgical approach is proposed, and, more specifically, 3–5 mL/kg/h for laparoscopic and 5–7 mL/kg/h for o procedures [49,50]. In all cases, rapid sequence induction (RSI) for anesthesia, combined with no face mask ventilation, remains the technique of choice for minimizing aspiration risk [51]. The risk for postoperative nausea and vomiting (PONV) should be evaluated with use of the Apfel score and antiemetic therapy should be administered as indicated [52]. Urinary catheterization and nasogastric tube placement should be avoided as much as possible and their use should be limited in cases where they are necessary [53]. In a cohort study by Yang et al. on patients undergoing emergency general surgery procedures, the overall rate of symptomatic venous thromboembolism was 3.9%, presenting a statistically significant difference compared to elective general surgery patients [54]. This highlights the importance of intensive care for thromboembolic prophylaxis, with the use of compression stockings or intermittent compression and low-molecular-weight heparin (LMWH) [55,56]. Opioid-sparing multimodal analgesia management provides effective postoperative pain relief, reduces postoperative need for opioids and opioid-associated adverse effects, and should prioritized in perioperative pain management [57]. In addition, local anesthetic infiltration of port sites and/or transabdominal plan blocks limit pain intensity and reduce the total doses of rescue analgesia [58].

Regarding the role of the surgical team in the intraoperative phase of the EUPEMEN protocol for AA management, surgeons should prefer minimally invasive approaches in most cases, taking into consideration personal experience and hospital resources. It has been proven that laparoscopic appendectomy is a safe option over open appendectomy, as it provides a series of benefits, including shorter hospitalization, decreased postoperative pain, early food tolerance, shorter time until return to activities, and lower surgical site infection rates [3,59]. In patients with complicated appendicitis, the placement of abdominal drains should be avoided as much as possible, since their use is associated with an increased risk of fistula formation, surgical site infections, bowel obstruction, paralytic ileus, and prolonged hospital stay [60,61,62]. Lastly, both surgical and nurse teams should implement perioperative care bundles to reduce surgical site infections (SSIs) [63].

#### 3.1.3. Immediate Postoperative Phase

The immediate postoperative phase of the EUPEMEN protocol for the management of AA takes place on the ward or in the resuscitation/intermediate care unit and is mainly conducted by the anesthesiologist, the surgeon, and the nurse. Postoperative hypothermia is encountered in up to 70% of noncardiac surgical procedures, predisposes patients to SSIs and cardiac dysfunction, and increases the need for transfusion [64]. The routine measurement of body temperature and active temperature maintenance, as well as routine oxygen saturation measurement aimed at the maintenance of FiO_2_ 0.5% for 2 h postoperatively, should be continued in the immediate postoperative phase to prevent hypothermia and hyposaturation. Perioperative restrictive fluid administration is recommended versus liberal fluid administration, since it has been associated with decreased rates of infectious, respiratory, and cardiovascular complications [65]. In addition, it is of utmost important for the whole team to ensure perioperative glycemia control of patients with diabetes mellitus according to the local hospital protocol, while for patients at risk of insulin resistance, such as obese and elderly patients, and for surgeries with a duration longer than sixty minutes, blood glucose concentration should be maintained below 180 mg/dL. Lastly, opioid-sparing multimodal analgesia is the key strategy for effective pain management, accelerated recovery, and hospital discharge [66].

The risk of symptomatic venous thromboembolism in patients undergoing appendicectomy is increased during the initial admission and after discharge, as well as in cases of open appendectomy [67,68]. Extended thromboprophylaxis with the use of compression stockings or intermittent compression and LMWH should be warranted according to the local hospital policy. Postoperatively, antibiotics should be administered therapeutically only in cases of complicated appendicitis and the regimen should be selected according to local hospital guidelines. For all other cases, antibiotic prophylaxis only is recommended [69]. Patients should be encouraged to sit up by two hours after surgery and begin ambulation eight hours after surgery with respect to night time sleeping hours [70]. Early oral feeding after appendectomy is safe and beneficial. Patients should be encouraged to start drinking four hours after surgery [71].

#### 3.1.4. Postoperative Days (POD) 1 and 2

The first postoperative days constitute a crucial part of enhanced recovery programs, with collaboration among the patient, surgeon, and nurse being fundamental for enhanced and uneventful recovery. From this time point, perioperative care takes place in the surgical ward. Numerous studies have proven that early discharge within 24 h after laparoscopic appendectomy in selected patients with uncomplicated appendicitis is a safe and cost-effective option, which is also associated with high patient satisfaction, when appropriate guidance and patient education are provided [72,73,74]. However, in most cases of complicated appendicitis, an increased length of hospital stay, even six times longer, is required in order to ensure the utmost patient safety and adequate pain control and to minimize morbidity and mortality rates [75]. Thus, the EUPEMEN guidelines for extended postoperative stay after the first 24 h after surgery should be reserved for cases of complicated appendicitis or for specific patient needs.

During this period, special emphasis should be placed on the early feeding of patients with oral diets containing semi-solid foods, since current data support the beneficial role of early feeding on postoperative recovery after appendectomy [76]. Solid foods could be offered on the second postoperative day if tolerated. It is well known that early mobilization with full ambulation and respiratory physiotherapy with a breathing device have a positive impact on post-discharge outcomes after emergency abdominal surgery [77]. A switch to opioid-sparing analgesia in peroral form should be considered for pain management [78]. The cessation of intravenous infusions should follow if patients are able to tolerate peroral fluid administration. Ongoing intravenous fluids should only be continued during the first postoperative days for patients with inadequate oral fluid intake. Patients after appendectomy might be vulnerable to postoperative thromboembolic events and special caution must be taken during the postoperative period [79]. Thromboembolic prophylaxis, including the use of compression stockings, intermittent pneumatic compression devices, and/or the administration of LMWH, should be continued postoperatively according to the local hospital policy. From the second postoperative day, patients should be evaluated for home-readiness and the fulfillment of early discharge criteria [80,81,82].

#### 3.1.5. Rest of Hospital Stay

The surgical and nursing teams are mainly responsible for the rest of the hospital stay for patients not discharged until the second postoperative day after appendectomy due to either patient-related factors or the severity of appendicitis. Key principles in enhanced recovery after surgery play an important role in improving perioperative outcomes even during this phase. These include early feeding, early mobilization combined with respiratory physiotherapy, pain management with peroral analgesia, thromboprophylaxis, and the continuation of antibiotics based on clinical, laboratory, or imaging findings. Patients’ safe discharge will be decided according to discharge criteria.

#### 3.1.6. Discharge

Discharge from hospital after a surgical procedure remains a fundamental stage of perioperative management, since unsolved discharge needs have been associated with poor perioperative outcomes and high readmission rates [83]. This phase encompasses, as in the majority of the previous phases, the surgeon, the nurse, and, additionally, the primary care physician, who may be called to assess the patient’s needs and queries postoperatively. Among the criteria that should be taken into consideration before discharge are postoperative laboratory blood tests, and, more specifically, the value of C-reactive protein, which should present a reduction of at least 50% prior to discharge [81]. Moreover, the general discharge criteria that should be fulfilled for safe discharge include no complications that are difficult to manage in an outpatient setting, no fever, adequacy of oral analgesia for pain management, and the acceptance and cooperation of the patient. In cases of complicated appendicitis, a short course of postoperative intravenous antibiotics has been proven to be non-inferior to a long course [84]. However, the continuation of antibiotic therapy in an outpatient setting should be decided in an individualized manner. Similarly, the risk of thromboembolic events will dictate the need for continued individualized thromboprophylaxis after discharge. Postoperative follow-up is suggested at 24 h after discharge in an outpatient setting in-person or via telephone, following a telemedicine model [83]. Further follow-up includes patient invitations for a check-up according to local hospital policy. Postoperative follow-up with a primary care provider has been associated with lower rates of hospital readmission, especially for high-risk surgical procedures or complicated cases [85]. Following this practice, home support visits by primary care physicians would be beneficial if needed.

## 4. Discussion

The concept of enhanced recovery programs, or otherwise fast-tracked surgery, was first introduced in 1997 in Northern Europe by Henrik Kehlet and colleagues, who established a research group aimed at improving postoperative outcomes after open colorectal surgical procedures [86]. This approach, having as a fundamental principle the minimization of surgical stress, has been adopted by an increasing number of surgeons over time and has been expanded in numerous kinds of surgical procedures [86]. Due to the significant postoperative improvement associated with fast-track surgery, in 2010, the “ERAS^®^ Society” was officially established, promoting a multimodal, multidisciplinary approach to the perioperative care of the surgical patient [87].

However, the implementation of the ERAS protocol still encounters numerous barriers and challenges, and its universal application and adoption are questioned [88]. Among them, organizational limitations, human and material resource scarcity, unwillingness to adopt new protocols, individual variability among physicians, and communication and coordination issues have been identified as substantial barriers to universal ERAS implementation [89]. Notably, the insufficient application of ERAS principles has led to poorer postoperative outcomes and decreased quality of healthcare services [88].

For this reason, the EUPEMEN project, a multicenter healthcare project funded by the European Union, was established, aiming to educate surgeons, anesthesiologists, and healthcare providers on the principles of enhanced recovery after surgery. The EUPEMEN protocol was produced by five partners with health and university profiles using data extracted by the clinical pathway of Recovery Intensification for optimal Care in Adult’s surgery (RICA), published by the Ministry of Health, Social Services, and Equality and the Aragon Health Sciences Institute [90]. The main objectives of the EUPEMEN project is the preparation of an educational project and the implementation of ERAS protocols in a homogeneous and standardized way. EUPEMEN embodies the collaboration of a multidisciplinary team, including the surgeon, the anesthesiologist, and the nurse, providing clear and evidence-based instruction for every stage of the perioperative process, for both elective and emergency settings, aimed at eliminating discrepancies and obscure points in perioperative care. The evaluation of the implementation results through the collection of data about perioperative outcomes of European Surgical patients and future comparison of previous short- and long-term clinical results with those that have arisen from the new program constitutes a secondary objective of the EUPEMEN initiative.

Accurately defining the severity of acute appendicitis is essential for guiding appropriate treatment strategies [20]. The severity assessment involves a combination of clinical evaluation, imaging studies, and intraoperative findings, each contributing to the differentiation between simple and complicated appendicitis [19].

The EUPEMEN protocol for the perioperative management of acute appendicitis represents a significant advancement in the standardization of care, particularly within the European context. While it shares foundational principles with the ERAS protocols, such as the emphasis on reducing surgical stress, promoting early mobilization, and minimizing opioid use, the EUPEMEN protocol offers more specific, evidence-based recommendations tailored to the management of acute appendicitis. Unlike ERAS, which provides a broad framework applicable to various surgical procedures, EUPEMEN integrates recent evidence and clinical expertise to address the unique challenges associated with appendicitis, especially in adult and elderly populations who often present with complex comorbidities. Furthermore, the EUPEMEN protocol distinguishes itself by incorporating multidisciplinary collaboration across multiple European healthcare systems, thus ensuring that its guidelines are adaptable and applicable across different clinical settings [90]. This contrasts with other national guidelines, which may focus on a narrower patient demographic or be limited by local clinical practices. For instance, while the ERAS guidelines are highly regarded for elective surgeries, their application in emergency situations such as acute appendicitis has been less clearly defined, often leading to variability in clinical practice. By comparison, the EUPEMEN protocol fills this gap by providing a comprehensive, step-by-step approach that is practical for real-world emergency settings.

The key difference between the existing ERAS protocols and the EUPEMEN project lies in the specificity and adaptability of our recommendations for acute appendicitis management within the European healthcare context, developed through multidisciplinary collaboration across Europe. While ERAS protocols provide a broad framework for enhancing recovery after various surgeries, the EUPEMEN protocol provides detailed, step-by-step recommendations specifically for acute appendicitis, addressing the unique challenges of emergency settings [91]. Additionally, it offers practical implementation tools, such as educational materials and multilingual manuals, to ensure consistent application across diverse healthcare environments. This approach equips clinicians with specialized, adaptable guidelines that are more directly applicable to their local contexts, ultimately improving patient outcomes. It should be highlighted that ERAS remains a broad, well-validated, and adaptable protocol suitable for a wide range of general surgery procedures, including for appendicitis [92]. However, the recently emerged EUPEMEN protocol offers more specialized, updated, and easier-to-implement guidelines, tailored to specific procedures, patient demographics, or clinical settings, and possesses the ability to address particular patient-specific needs more effectively than a one-size-fits-all approach, through the harmonic elaboration of numerous healthcare specialists. The EUPEMEN protocol was built upon ERAS by integrating newer evidence, addressing specific gaps, and providing more detailed guidance for particular surgical issues. Through its step-wise approach for every stage of perioperative care, it provides clearer, more detailed guidance for perioperative care, thus increasing the rates of successful adoption, compliance, and implementation.

In comparison to the World Society of Emergency Surgery (WSES) guidelines, which are widely recognized for their comprehensive recommendations on the diagnosis and management of acute appendicitis, the EUPEMEN protocol offers a more focused approach on perioperative care, emphasizing enhanced recovery principles [93]. The WSES guidelines primarily address the overall management of acute appendicitis, including diagnostic algorithms, the timing of surgery, and antibiotic therapy. While these guidelines are crucial for guiding clinical decision-making in emergency settings, they are less detailed in terms of perioperative care strategies aimed at reducing postoperative morbidity and enhancing recovery [93]. The EUPEMEN protocol complements the WSES guidelines by providing a structured, multidisciplinary approach to perioperative management, with specific recommendations on preoperative, intraoperative, and postoperative care tailored to the needs of adult and elderly patients. This focus on the perioperative pathway, combined with the integration of ERAS principles, allows the EUPEMEN protocol to address aspects of patient care that are not as extensively covered in the WSES guidelines, thereby offering a more comprehensive framework for optimizing outcomes in patients undergoing appendectomy for acute appendicitis.

The EUPEMEN project introduces several key determinants in clinical practice that were not fully addressed and organized in the setting of the guidelines so far. First of all, the EUPEMEN project offers a tailored approach specifically for the perioperative management of acute appendicitis, addressing unique aspects of this condition that are not comprehensively covered by broader ERAS protocols. This includes detailed guidance on the timing of interventions and managing complications specific to acute appendicitis. Second, the emphasis on multidisciplinary coordination among surgeons, anesthesiologists, nurses, and allied health professionals is crucial for optimizing care, but often lacks structured guidance in existing protocols. The EUPEMEN project provides this structure, ensuring that all relevant specialists are effectively collaborating to enhance patient outcomes. Third, the EUPEMEN project is designed to be adaptable to various European healthcare systems, accounting for differences in resources, practices, and patient populations. This localized approach ensures that clinicians can apply the best standards of care in ways that are specifically suited to their healthcare environment, a nuance that may not be captured by existing, more generalized protocols. Finally, the EUPEMEN project includes practical implementation tools such as educational resources, online courses, and multilingual manuals, which help bridge the gap between theoretical knowledge and practical application. These tools are essential for ensuring that the guidelines are not only understood, but also effectively integrated into daily clinical practice.

It must be recognized that during the production of the protocol, several issues were encountered where it was challenging to achieve complete agreement among the experts. These differences reflect the varying levels of evidence available, as well as the influence of local practices and resource availability in different healthcare systems. In these cases, the panel engaged in thorough discussions, weighing the pros and cons of each approach. Where strong evidence was available, this guided the final recommendations. In areas where evidence was less clear or where practice variability is common, the protocol provides flexible guidelines that can be adapted based on specific patient needs and local contexts. This approach ensures that the EUPEMEN protocol remains both evidence-based and practically applicable.

The potential for the protocol developed by the five European experts to be adopted universally within their institutes—and beyond—depends on several key factors. Its adaptability allows for customization to meet the specific needs of different institutions, which can facilitate broader application. To gain wider acceptance, the protocol would benefit from further evidence-based validation through multicenter studies, audits, and real-world application, demonstrating its effectiveness and safety in diverse settings. Additionally, endorsements from national and international professional bodies and surgical societies can enhance its credibility and support its integration as a standard guideline.

In terms of the universal adoption and application of the EUPEMEN protocol for AA, several obstacles may arise. Variability in clinical practice, differences in resources and expertise, and potential resistance to change could impact its uniform application. Overcoming these challenges requires developing customized implementation plans, establishing comprehensive education and training programs for healthcare professionals, and conducting pilot studies to gather feedback and refine the protocol. Engaging with stakeholders and emphasizing the protocol’s evidence-based benefits could also help address resistance and ensure successful integration into clinical practice.

In conclusion, while the EUPEMEN protocol builds upon the strengths of existing guidelines, it offers a more specialized approach that is both versatile and deeply informed by the collective expertise of European surgical, anesthetic, and nursing teams. This unique combination of specificity and adaptability positions the EUPEMEN protocol as a valuable tool in enhancing perioperative outcomes for patients with acute appendicitis, particularly within the diverse healthcare environments of Europe.

## 5. Conclusions

The primary purpose of this manuscript is to present a consensus-based protocol for the perioperative management of acute appendicitis in adult patients. The protocol was developed through the synthesis of clinical experience and expert opinion from a multidisciplinary team across various European healthcare settings, addressing the practical challenges faced in real-world scenarios. We recognize that acute appendicitis presents a spectrum of clinical manifestations, from mild cases managed conservatively with antibiotics to severe cases requiring surgical intervention. Our protocol is designed to be adaptable to these varying clinical scenarios, providing tailored recommendations that can be implemented flexibly across different settings. While our approach does not aim to generalize findings through systematic data collection, it offers valuable guidance rooted in current clinical practices, aiming to standardize and enhance perioperative care in diverse healthcare environments.

The EUPEMEN project, arisen by the theoretical fundamentals and the clinical expertise of a research group from four different European countries, is a patient-centered process that aims to establish the universal application of ERAS principles in order to achieve the best possible perioperative outcomes, both for elective and emergency surgical procedures. In particular, the EUPEMEN protocol for AA leads to faster postoperative recovery, reduced length of hospital stay, and improved postoperative morbidity and mortality metrics through the coordination of diverse specialists.

## Data Availability

The data presented in this study are available at https://eupemen.eu/, accessed on 1 November 2024).
